# Relative Contributions of Vibrio Polysaccharide and Quorum Sensing to the Resistance of *Vibrio cholerae* to Predation by Heterotrophic Protists

**DOI:** 10.1371/journal.pone.0056338

**Published:** 2013-02-18

**Authors:** Shuyang Sun, Staffan Kjelleberg, Diane McDougald

**Affiliations:** 1 Centre for Marine Bio-Innovation, School of Biotechnology and Biomolecular Sciences, The University of New South Wales, Sydney, New South Wales, Australia; 2 Singapore Centre on Environmental Life Sciences Engineering, Nanyang Technological University, Singapore, Singapore; 3 School of Biological Sciences, Nanyang Technological University, Singapore, Singapore; 4 Advanced Environmental Biotechnology Centre, Nanyang Environment and Water Institute, Nanyang Technological University, Singapore, Singapore; Charité-University Medicine Berlin, Germany

## Abstract

Protozoan grazing is a major mortality factor faced by bacteria in the environment. *Vibrio cholerae,* the causative agent of the disease cholera, is a natural inhabitant of aquatic ecosystems, and its survival depends on its ability to respond to stresses, such as predation by heterotrophic protists. Previous results show that grazing pressure induces biofilm formation and enhances a smooth to rugose morphotypic shift, due to increased expression of Vibrio polysaccharide (VPS). In addition to negatively controlling *vps* genes, the global quorum sensing (QS) regulator, HapR, plays a role in grazing resistance as the Δ*hapR* strain is efficiently consumed while the wild type (WT) is not. Here, the relative and combined contributions of VPS and QS to grazing resistance were investigated by exposing VPS and HapR mutants and double mutants in VPS and HapR encoding genes at different phases of biofilm development to amoeboid and flagellate grazers. Data show that the WT biofilms were grazing resistant, the VPS mutants were less resistant than the WT strain, but more resistant than the QS mutant strain, and that QS contributes to grazing resistance mainly in mature biofilms. In addition, grazing effects on biofilms of mixed WT and QS mutant strains were investigated. The competitive fitness of each strain in mixed biofilms was determined by CFU and microscopy. Data show that protozoa selectively grazed the QS mutant in mixed biofilms, resulting in changes in the composition of the mixed community. A small proportion of QS mutant cells which comprised 4% of the mixed biofilm biovolume were embedded in grazing resistant WT microcolonies and shielded from predation, indicating the existence of associational protection in mixed biofilms.

## Introduction

Predation by heterotrophic protists has been identified as one of the key environmental pressures faced by bacteria and thus, the survival and persistence of bacteria depends on their resistance to grazing pressure. Due to the long history of coexistence of bacteria and bacterivorous predatory protists, bacteria have evolved a number of grazing resistance strategies [1]. *Vibrio* (*V.*) *cholerae*, the causative agent of cholera is a natural inhabitant of aquatic ecosystems, and its long-term persistence in the environment is dependent on resistance to predation [2]–[4]. Biofilm formation and aggregation has been suggested to be important for survival of most microorganisms [5]–[7] and to contribute to the environmental persistence of *V. cholerae* [8]–[10]. Indeed, simple filtration of water through used sari cloth was shown to remove up to 99% of *V. cholerae* cells, by retaining the plankton and associated *V. cholerae* [11] and this practice was demonstrated to reduce cholera infection by 48% [12]. We have shown previously that *V. cholerae* exhibits an increase in biofilm and/or microcolony formation in response to protozoan grazing, and that these biofilms are grazing resistant, while planktonic cells are rapidly eliminated [13]–[14].

Vibrio polysaccharide (VPS) facilitates the attachment of *V. cholerae* cells to a surface and constitutes a major component of *V. cholerae* biofilm matrix [15]. Furthermore, VPS is responsible for the increased resistance of biofilm cells to a variety of stresses, including chlorine [15]–[17], acid [18], osmotic and oxidative stress [19], anti-bacterial serum [17], phage [20], and sodium dodecyl sulphate [21]. Predation by phagotrophic protists stimulates VPS production, resulting in biofilm formation and a smooth rugose morphologic shift [13]. Similarly, a mucoid strain of *Pseudomonas aeruginosa* which over-expresses exopolymeric substances (EPS) has increased resistance to predation [22]. Therefore, VPS may be an important factor in protecting *V. cholerae* from protozoan grazing, independent of the formation of complex structures.

The structural genes required for VPS production reside in two carbohydrate biosynthesis operons (*vpsA-K* and *vpsL-Q*) which are positively regulated by VpsR and VpsT, but repressed by the quorum sensing (QS) system response regulator, HapR [23]. There are two signalling pathways in *V. cholerae* that are co-ordinately regulated to control the expression of the master regulator, HapR [24]. In addition to the regulation of VPS synthesis, HapR also regulates the expression of anti-protozoal activity produced by *V. cholerae* biofilms [13]. The regulation of anti-protozoal activity in high cell density biofilms by QS may be a general feature that allows for the concentration of anti-protozoal factors not possible for cells in the planktonic phase.

Biofilm formation is a dynamic process that is characterised by sequential stages of biofilm development and maturation [25]. Different protozoa are specialised at colonising biofilms at different stages of the biofilm life cycle. For example, many protozoa are suspension feeders, ingesting bacteria in the planktonic phase, while others are surface feeders and colonise either early or late biofilms [26]. In the case of *P. aeruginosa* PAO1, EPS production and QS-regulated toxin production protect early and late biofilms respectively against early and late biofilm colonisers [27]. Since VPS and QS are involved in *V. cholerae* biofilm formation, their contributions to grazing resistance may also vary in importance at the different stages of the biofilm life cycle.

In this study, the relative and combined contributions of VPS and QS in grazing defence of *V. cholerae* were determined using VPS, QS and double deficient mutants. The differences in resistance gained from VPS and QS through biofilm development were also investigated by exposing *V. cholerae* early and late biofilms to protozoan grazing. Finally, we addressed the competitive fitness of the QS mutant and wild type (WT) strains of *V. cholerae* A1552 in mixed biofilms exposed to predation.

## Materials and Methods

### Bacterial and Protozoal Strains and Growth Conditions

Organisms used in this study are listed in Table 1. Bacterial strains were routinely maintained in Luria-Bertani (LB) broth and on agar plates supplemented, as appropriate, with chloramphenicol (34 µg ml^−1^ for *E. coli* and 5 µg ml^−1^ for *V. cholerae*), ampicillin (50 µg ml^−1^) and polymixin B (40 µg ml^−1^). *Rhynchomonas* (*R.*) *nasuta*, a surface feeding flagellate, was isolated from the Sydney Institute of Marine Science [14]. To eradicate indigenous bacteria, the *R. nasuta* culture was treated with a combination of antibiotics (ampicillin, gentamycin, kanamycin, spectomycin, streptomycin and tobramycin at 150 µg ml^−1^) and serial diluted for over many generations. *R. nasuta* was routinely grown on heat-killed *P. aeruginosa* PAO1 (final concentration 10^7^ cells ml^−1^) in 50% NSS medium (NSS is an artificial seawater medium which contains 17.6 g NaCl, 1.47 g Na_2_SO_4_, 0.08 g NaHCO_3_, 0.25 g KCl, 0.04 g KBr, 1.87 g MgCl_2_•6H_2_O, 0.45 g CaCl_2_•2H_2_O, 0.01 g SrCl_2_•6H_2_O and 0.01 g H_3_BO_3_ in one liter of distilled water, 50% NSS contains half of the salts in NSS medium) [28] statically at room temperature (RT). *Acanthamoeba* (*A.*) *castellanii,* a surface feeding amoeba obtained from the American Type Culture Collection (ATCC30234), was maintained using the same procedure as for *R. nasuta*, but incubated at 30°C. Protozoa were subcultured three days prior to grazing experiments and enumerated microscopically using a haemocytometer. Aliquots were plated on LB agar to rule out bacterial contamination before grazing experiments.

**Table 1:** Bacterial strains, protozoa and plasmids used in this study.

Strain/Plasmid	Properties	Reference or origin
**Bacterial strains**		
*V. cholerae* A1552	O1 El Tor, Inaba, Rif^r^	[15]
*V. cholerae vpsA*	A1552, Δ*vpsA*	[15]
*V. cholerae vpsL*	A1552, Δ*vpsL*	[15]
*V. cholerae hapR*	A1552, *hapR*::pGP704, Ap^r^	[47]
*V. cholerae vpsA/hapR*	A1552, Δ*vpsA hapR*::pGP704-*cat*,Cm^r^	This study
*V. cholerae vpsL/hapR*	A1552, Δ*vpsL hapR*::pGP704-*cat*,Cm^r^	This study
*V. cholerae* A1552 GFP	A1552, mTn*7*-GFP, Gm^r^	[53]
*V. cholerae hapR* RFP	*hapR*, mTn*10*-DsRedExpress, Ap^r^ Km^r^ Cm^r^	This study
*E. coli* S17-1 λ*pir*	*recA thi pro* r_K_^-^ m_K_^+^ *RP4*::2-Tc::MuKm Tn*7* Tp^r^ Sm^r^ λ*pir*	[54]
**Protozoa**		
*Rhynchonomas nasuta*	Wild Type	[14]
*Acanthamoeba castellanii*	Wild Type	ATCC 30234
**Plasmids**		
pGP704.*hapR*.*cat*	pGP704 [55] containing 0.3 kb internal region of *hapR* disrupted by 0.9 kb *cat* from pLG401(Lynn Gilson, University of Hawaii)	This study
pKKS07	Km^r^, Ap^r^, Cm^r^, pBLS180 [56] containing *dsred* and *cat* from pBK-miniTn*7dsred* [57]	Krager Koh, Nanyang Technological University

*V. cholerae* double mutant strains were constructed by null mutation of the *hapR* gene in Δ*vpsA* and Δ*vpsL* strains. The suicide plasmid pGP704.*hapR.cat* was introduced by conjugation with *E. coli* S17-1 λ*pir* [21], [29]. The *V. cholerae* Δ*hapR* strain was tagged with red fluorescent protein (RFP) by transposon mutagenesis using DsRedExpress introduced on pKKS07 (constructed by Dr. K. KOH, Singapore Centre on Environmental Life Sciences Engineering, Singapore).

### Selective Grazing Assay

To test the relative grazing resistance of various strains of *V. cholerae,* the selective grazing assay was performed as previously reported with modifications [30]. Briefly, overnight cultures of *V. cholerae* strains were adjusted to 10^7^ cells ml^−1^ and subsequently inoculated by streaking 1 cm from the centre of Vaatanen nine salts solution (VNSS, which contains 1 g bacteriological peptone, 0.5 g yeast extract, 0.5 g D-glucose, 0.01 g FeSO_4_•7H_2_O and 0.01 g Na_2_HPO_4_ in one liter of 50% NSS) [28] agar plate outward with eight replicates. *A. castellanii* were enumerated by microscopy and a total of 6×10^4^ cells in 15 µl of 50% NSS were added to the centre of each plate at the beginning of the experiment. Plates were incubated at RT for 15 days. The distance of the grazing front of each streak was measured to determine the grazing resistance.

### Biofilm Grazing Assay

To assess the grazing resistance of *V. cholerae* biofilms, batch experiments were performed in 24-well tissue culture plates as previously reported with modification [27]. Early biofilm grazing assays were performed by simultaneous addition of *V. cholerae* strains and protozoa to the microtitre plates and the biofilm biomass was measured after three days. Overnight cultures of *V. cholerae* were diluted to 2×10^5^ cells ml^−1^ in VNSS medium. Five hundred µl of diluted *V. cholerae* cultures were added to 4×10^4^ cells ml^−1^ of *R. nasuta* (which feeds on single attached cells) and *A. castellanii* (which feeds on clumps of cells) in 500 µl of 50% NSS. The plates were incubated for three days at RT with shaking at 60 rpm. Late biofilm grazing assays were performed by adding 10^5^ cells ml^−1^ of *V. cholerae* into the wells and incubating at RT with shaking at 60 rpm for three days to allow the biofilms to establish, after which the medium was replaced with fresh medium containing 2×10^4^ cells ml^−1^ of *R. nasuta* and *A. castellanii*. Generally, four replicates were applied for each treatment.

### Quantification of Biofilm Biomass

The biomass of *V. cholerae* biofilms was quantified by a crystal violet (CV) staining assay [31]. Briefly, the aqueous phase was removed from each well, and planktonic cells were removed by washing the biofilm three times with 1 ml of 50% NSS. One ml of CV (0.3% w/v) was added to each well, incubated for 20 min and rinsed three times with 1 ml of 50% NSS before the bound CV was solubilised in 1 ml of 95% ethanol. The absorbance (490 nm) was determined with a plate reader (Wallac, Gaithersburg, MD).

### Determination of the Relative Abundance of V. cholerae Strains in Mixed Biofilms

Biofilm grazing assays were prepared as described above. The aqueous phase was removed from each well, and planktonic cells were thoroughly washed from the surface with 50% NSS. To determine the relative abundance of *V. cholerae* strains in mixed biofilms, the biofilms were scraped off with a sterile spatula from the surface of the well and resuspended in 1 ml of 50% NSS medium. The suspended biofilms in the microtitre plates were moderately sonicated (Unisonics FXP12MH, Brookvale, Australia) for 1 min to reduce cell aggregates which were verified by microscopy. *V. cholerae* cells from the mixed biofilms were diluted and plated on LB agar plates for determination of total CFU and on LB agar plates supplemented with 50 µg ml^−1^ ampicillin for enumeration of the Δ*hapR* strain.

### Confocal Laser Scanning Microscopy and Image Analysis

To visualise the spatial arrangement of the mixed strain biofilms, confocal laser scanning microscopy (CLSM) was used. *V. cholerae* A1552 GFP and Δ*hapR* RFP were exposed to protozoan grazing in biofilm grazing assays. After protozoan grazing, the mixed biofilms were imaged by CLSM (FV1 000, Olympus, Japan) under 400× magnification. The percentage of each strain in mixed biofilms was calculated using biovolumes determined by IMARIS (http://www.bitplane.com/go/products/imaris).

### Statistical Analysis

One-way ANOVAs were used for the analysis of selective grazing assays and Tukey’s Multiple Comparison Test provided the post-hoc comparison of means. Two-tailed student’s t-tests were used to compare means, e.g. the biofilm of *V. cholerae* strains under protozoan grazing versus non-grazed controls.

## Results

### Selective Grazing of VPS and QS Mutants

In order to determine the relative contribution of VPS and the QS-regulated anti-protozoal factors produced by *V. cholerae,* we investigated the grazing resistance of the WT strain, mutants in the first genes of the two VPS operons, *vpsA* and *vpsL*, and a mutant in the transcriptional regulator of the QS system, *hapR*. In addition, strains mutated in both VPS and QS genes were generated and compared to the above strains to elucidate the combined effect of VPS and QS in grazing resistance.

**Figure 1:**
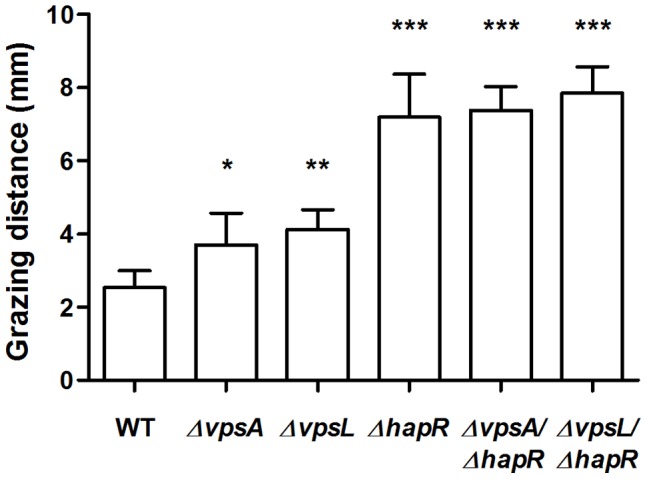
Selective grazing assays of *V. cholerae* A1552, VPS, QS and VPS/QS mutants. Strains were grown on VNSS agar plates incubated for 15 days at RT. The relative grazing resistance is reflected by the distance from the grazing starting position to the grazing front. Error bars represent standard deviation. The experiment was run in replicates of eight and repeated three times.

In a selective grazing assay where the predator has a choice of prey strains, the most palatable strains will be preferentially consumed and thus the line of inoculation will be reduced more than less desirable prey. In the selective grazing assay done here, the WT strain was the least grazed, and thus the most resistant to *A. castellanii* predation (Fig. 1). The two VPS structural gene mutants, Δ*vpsA* and Δ*vpsL,* were grazed more than the WT (p<0.05), indicating that VPS provides protection from predation by amoeba. All of the QS mutants, including the two VPS/QS double mutants, were grazed significantly more than the WT strain and the two VPS mutant strains (p<0.001), demonstrating the importance of QS-regulated anti-protozoal activity in grazing resistance. The differences in grazing distances between the QS single and QS/VPS double mutants were not significant (Fig. 1).

### Grazing Resistance of Early and Late Biofilms

**Figure 2:**
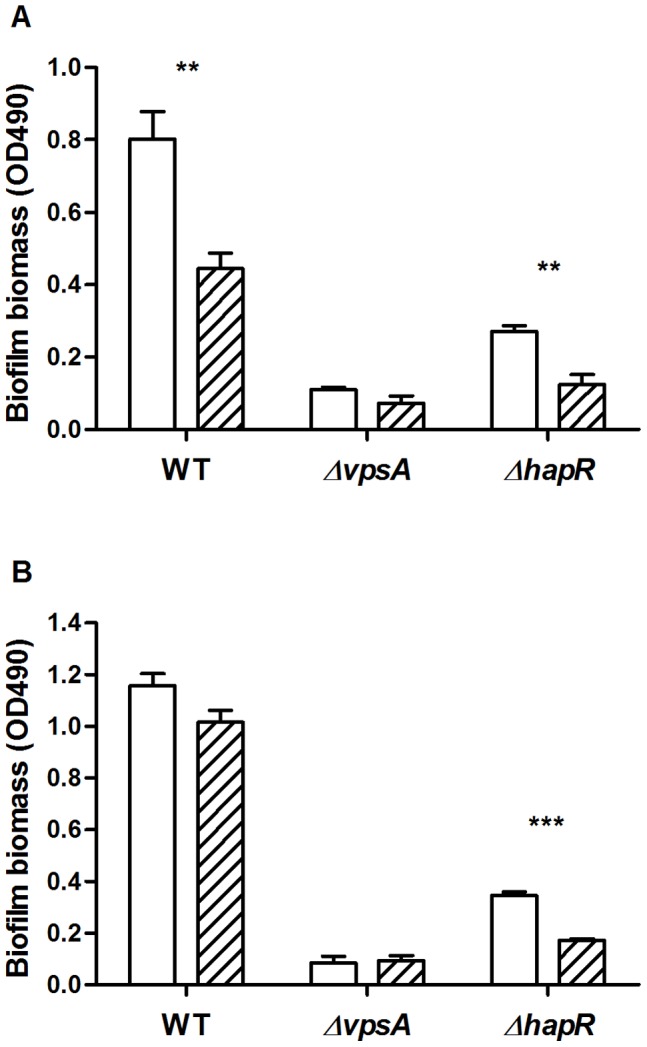
Biofilm grazing assays of *V. cholerae* A1552, Δ*vpsA* and Δ*hapR* strains. Grazing resistance of early (A) and late (B) biofilms was determined by comparing the biofilm biomass of *V. cholerae* strains in the absence (open) and in the presence (hatched) of *R. nasuta* and *A. castellanii*. Error bars represent standard deviation. The experiment was run in replicates of four and repeated twice.

It is known that there is a succession pattern of predators that colonise biofilms at various stages of biofilm development [26]. Thus, the contributions of VPS and QS to grazing resistance may also vary at the different stages of the biofilm life cycle. To determine the differences in the contributions of VPS and QS to grazing resistance, *V. cholerae* biofilms in early and late developmental stages were challenged with *R. nasuta* and *A. castellanii*. The two grazers used here did not interfere with each other when co-incubated (data not shown). In the early biofilm assay, initial colonisation of the surface and subsequent microcolony formation of *V. cholerae* occurred in the presence of grazing pressure (Fig. 2A). Compared to non-grazed controls, the WT and Δ*hapR* strains were grazed by 45% and 54% respectively (p<0.01), while the Δ*vpsA* strain was not significantly grazed.

To explore the grazing resistance of late stage biofilms, the biofilms were pre-grown for three days prior to exposure to the protozoa. Results show that grazing did not significantly reduce the biomass of the WT and Δ*vpsA* biofilms (Fig. 2B). In contrast, the biofilm biomass of the Δ*hapR* strain was reduced by 50% (p<0.001). The contribution of VPS to grazing resistance observed in the selective grazing assays was not apparent in the biofilm grazing assays where the biomass of the Δ*vpsA* biofilms in early and late stages was not significantly reduced by grazing pressure. The unexpected lesser VPS contribution to biofilm grazing resistance is probably due to the fact that the Δ*vpsA* strain forms very little biofilm biomass under the laboratory conditions used in this experiment, and that the CV staining method is not very sensitive. The WT and Δ*hapR* strains were grazed to the same extent in early biofilms, indicating there was no QS-related grazing resistance at this stage (Fig. 2A). In contrast, late biofilms of the WT strain displayed QS dependent grazing resistance, as deduced by the comparative decrease in biomass of the Δ*hapR* strain (Fig. 2B).

### Competitive Fitness of WT and ΔhapR Strains in Mixed Biofilms Under Grazing Pressure

**Figure 3:**
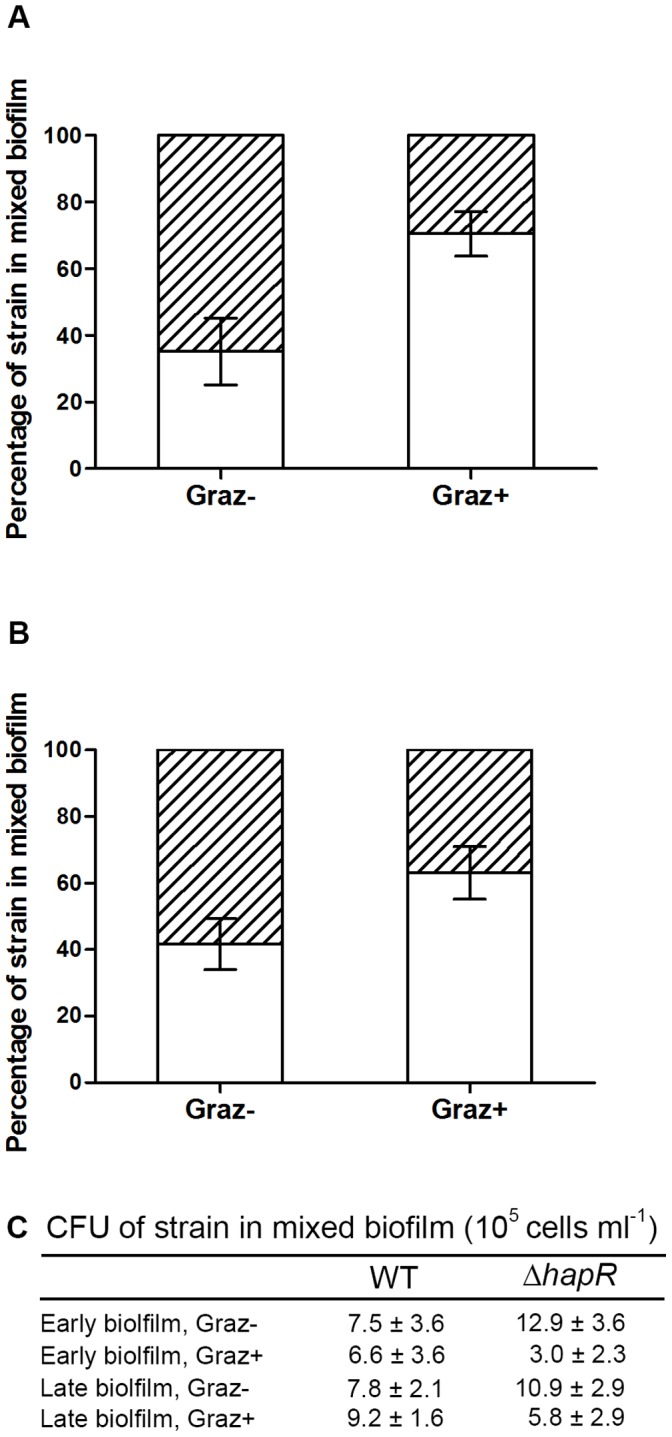
Biofilms of mixed *V. cholerae* A1552 (open) and Δ*hapR* strains (hatched) were exposed to predation. The relative fitness of each strain in early (A) and late (B) biofilms of mixed *V. cholerae* strains was measured by enumeration of CFU (C). Error bars represent standard deviation. Experiments were run in replicates of four and repeated twice.

To investigate the effect of protozoan grazing on mixed biofilms of the grazing resistant *V. cholerae* WT and the grazing sensitive Δ*hapR*, the strains were mixed at a 1∶1 ratio and exposed to predation by *R. nasuta* and *A. castellanii* in early and late stages of biofilm development. The relative abundance of each *V. cholerae* strain was determined by CFU, obtained by plating on LB (for total CFU) and on LB plates supplemented with ampicillin (50 µg ml^−1^, for the Δ*hapR* CFU). Protozoan grazing on the mixed biofilm community resulted in a significant reduction in the proportion of Δ*hapR* cells, from 65% to 30% in the early biofilm assays (p<0.01) (Fig. 3A) and from 58% to 37% in the late biofilm assays (p<0.01) (Fig. 3B).

**Figure 4:**
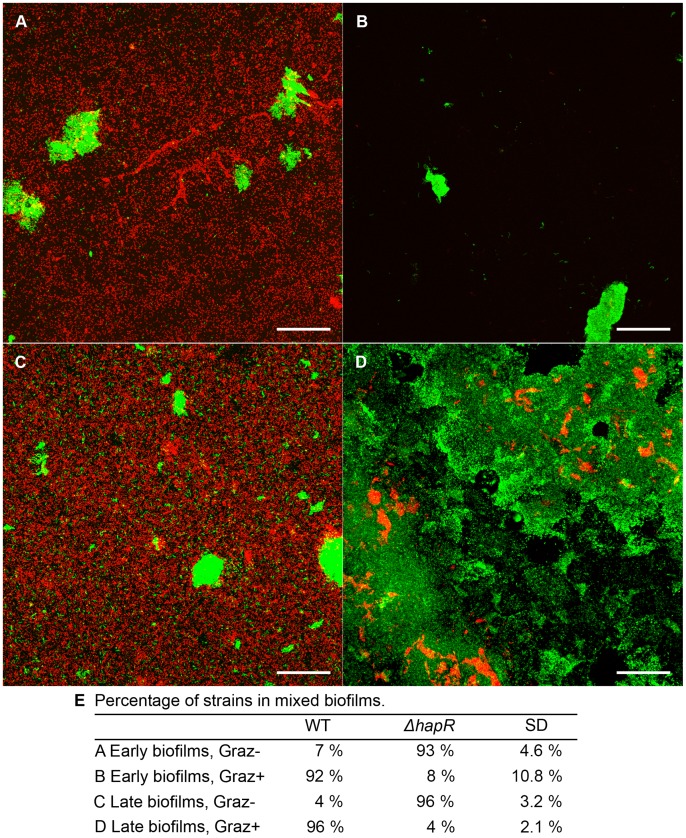
Confocal laser scanning microscopic images of mixed biofilms of *V. cholerae* A1552 and Δ*hapR* strains. Three dimensional images of early (A, B) and late (C, D) biofilms in the absence (A, C) and presence (B, D) of predation. (Magnification, ×400; scale bar, 50 µm.) The percentage of each strain in mixed biofilms was calculated using biovolumes determined by IMARIS (E). Experiments were run in replicates of four and repeated twice.

To further investigate the effect of protozoan grazing on mixed biofilms, the WT strain was tagged with GFP and the Δ*hapR* with RFP, and the three dimensional architecture of the mixed biofilms was determined by CLSM imaging and analysis using IMARIS. In non-grazed early biofilm controls, the Δ*hapR* strain dominated the biofilm, comprising 93% of the biofilm biovolume, forming a flat unstructured biofilm, while the WT was localised mainly in microcolonies (Fig. 4A). In the presence of grazing pressure, the WT microcolonies were resistant to grazing, comprising 92% of the biovolume, while most of the Δ*hapR* biomass was consumed (p<0.001) (Fig. 4B).

Late stage mixed biofilms, in the absence of protozoan grazing, were similar to early stage biofilms as the Δ*hapR* strain was predominant, comprising 96% of the biovolume (Fig. 4C). When the late mixed biofilms were grazed by *R. nasuta* and *A. castellanii*, the WT microcolonies were protected from predation, comprising 96% of the biovolume, while the Δ*hapR* cells were selectively removed (p<0.001) (Fig. 4D). Cells of the Δ*hapR* strain were observed in the middle of WT microcolonies, indicating that the WT population offered associational protection for Δ*hapR* cells. In addition, protozoan grazing on late biofilms also enhanced the biofilm formation of the WT strain, as the biofilm biovolume was 11 fold higher and the microcolonies were more differentiated than microcolonies formed by the WT in non-grazed controls (Fig. 4C and D).

## Discussion

### Relative Contribution of VPS and QS-regulated Phenotypes to Grazing Resistance of V. cholerae

In this study, we investigated the role of VPS and QS in grazing resistance by exposing *V. cholerae* WT and mutant strains to amoeboid and flagellate grazers. The choice of predators was limited by the availability of axenic cultures of protozoa. Although *A. castellanii* and *R. nasuta* are representive surface feeders which have been used widely in studies of protozoan grazing on bacterial biofilms [13]–[14], [22], [32]–[34], the findings obtained here might not be applicable to other protozoa with different grazing behaviours.

Protozoan grazing has been shown to stimulate *V. cholerae* biofilm formation and a smooth to rugose morphotypic shift by enhancing VPS production [13]. Here, the selective grazing assays using VPS deficient mutants revealed that VPS production contributed to resistance to predation by *A. castellanii*, where Δ*vpsA* and Δ*vpsL* strains were grazed significantly more than the parent WT strain (Fig. 1). In contrast, the contribution of VPS to grazing resistance was not apparent in the 24-well microtitre plate biofilm grazing assays where CV staining was used to determine biomass (Fig 2), reflecting the lower resolution by this method compared to that of selective grazing assay. VPS may act to protect encased cells by preventing access of the predator, or increase aggregation of cells where aggregates may exceed the optimal size range of prey, as bacterial cell size is a key selective parameter for protozoan feeding behaviour [35]. Furthermore, VPS as the main component of the extracellular matrix of *V. cholerae* biofilms may allow the retention of bioactive molecules secreted by the biofilm [13], effectively increasing the anti-protozoal activity of the biofilm. Indeed, we have shown that biofilms of *V. cholerae* incubated in the marine environment are grazing resistant, further confirming the ecological relevance of biofilms as a mechanism for grazing resistance [14]. Additionally, VPS may protect engulfed cells from subsequent digestion, as VPS has been reported to protect *V. cholerae* from acidity, allowing passage through the human digestion system [18].

Results presented here show that the QS deficient mutant, Δ*hapR*, was grazed much more than the WT and the VPS deficient mutants, indicating that grazing resistance provided by QS-regulated phenotypes is relatively more important than VPS expression. The fact that the differences between the grazing resistance of the QS mutant and the QS/VPS double mutants were not significant indicates that the combined protection from VPS and QS in grazing resistance is not significantly different from the protection afforded by expression of QS-regulated genes alone. Therefore, the QS-regulated activity has a significantly larger role than VPS production in protection against predation by *A. castellanii* in this study.

A role for QS-regulated mechanism in grazing resistance has been previously reported. *Chromobacterium violaceum* produces violacein at high cell density under the regulation of QS [36]. This compound is produced by a range of bacteria and has been shown to be toxic to a number of protozoa, resulting in apoptosis [37]–[38]. *P. aeruginosa* PAO1 biofilms produce a QS-regulated compound that is toxic to flagellates [22] and *P. fluorescens* CHA0 secretes anti-protozoal secondary metabolites under QS regulation [39]–[40]. We have previously reported that *V. cholerae* biofilms secrete a QS-regulated anti-protozoal factor [13]. In addition, the extracellular protease PrtV [41] and inhibitory factor VasX delivered by the type VI secretion system [42]–[44] are also regulated by QS and involved in grazing resistance of *V. cholerae*, further indicating the role of QS in grazing resistance. Therefore, production of QS-regulated anti-protozoal activity may be a common grazing resistance strategy that has evolved in bacteria.

### Contribution of QS to Grazing Resistance at Different Stages of Biofilm Development

The contributions of VPS and QS to grazing resistance in early and late biofilms were investigated in this study (Fig. 2). The contribution of VPS was not apparent in early or late biofilms, probably due to the fact that there was very little biofilm formed by the Δ*vpsA* mutant and that the resolution using the CV staining method is low. With respect to the contribution of QS in grazing resistance, early biofilms formed by *V. cholerae* WT and Δ*hapR* strains were grazed effectively, indicating that there is no QS-regulated grazing resistance expressed in the process of initial colonisation and microcolony formation. In mature biofilms, however, the WT was not significantly affected by predation while the Δ*hapR* strain was largely eliminated, demonstrating pronounced QS-mediated grazing resistance provided by mature biofilms. The QS dependent grazing resistance in mature rather than early biofilms is consistent with the role of QS in the biofilm development process, where QS is activated only when the cell density in the microcolonies reaches a threshold [24]. Similarly, *P. aeruginosa* biofilms have been shown to express a QS-regulated toxicity, in the late stages of the biofilm life cycles [27]. The regulation of anti-predation defences by QS may be a common theme for biofilms as these biofilms are sessile and subject to intense grazing pressure in the environment. The secretion of defence chemicals at the late stages of biofilm development when the cell density is high would help to ensure that effective concentrations of anti-predation compounds are produced.

### Protozoan Grazing on Mixed V. cholerae Strain Biofilms

The CFU data and the CLSM analysis revealed that the Δ*hapR* strain dominated the mixed biofilms, although the percentages differed (65% Δ*hapR* in early and 58% in late biofilms for CFU, Fig. 3; 93% and 96% for CLSM of early and late biofilms respectively, Fig. 4). The discrepancy between two approaches may be due to aggregation of *V. cholerae* Δ*hapR* cells, which results in an under estimation of the CFUs. It is known that the Δ*hapR* strain tends to aggregate due to increased VPS production [18]. Similarly, while there is no evidence that cells became viable but non-culturable in the grazing assays, this is also a possibility as *V. cholerae* is known to enter this state under nutrient starvation [45]. Nonetheless, both the CFU and CLSM measures demonstrated the same trend where the Δ*hapR* strain dominated the mixed biofilms in the absence of protozoan grazing. It has been reported previously that the *V. cholerae* Δ*hapR* strain exhibits a growth advantage over the QS positive strain when both strains are grown in biofilms [46].

The proportion of the WT increased in mixed biofilms when protozoan grazing was introduced as assessed by both CFU and CLSM analysis. CLSM image analysis revealed significant selective grazing effects on the Δ*hapR* strain. In contrast, the microcolonies formed by the WT demonstrated grazing resistance, and even enhanced growth in late stage mixed biofilms. Such selective grazing behaviour has been reported for predation on *P. fluorescens*, where the QS deficient strain was selectively removed from mixed biofilms formed with the parent WT [40].

Selective removal of the QS mutant strain in mixed biofilms indicates that protozoa are able to distinguish *V. cholerae* WT and Δ*hapR* cells, potentially by differences in cell surface characteristics. For example, since VPS production is regulated by QS [18], [46]–[47], the VPS composition of WT and Δ*hapR* cells may be different. It has been reported that mannose, a common sugar component of EPS, including VPS, can be recognised specifically by carbohydrate binding proteins on the cell surface of *A. castellanii* [48]. Also, O-antigen variability of *Salmonella enterica* has been shown to affect grazing efficiency of amoeba [30]. In addition, the hydrophobicity of the bacterial cell surface may affect prey capture by protozoa, when *Prochlorococcus* cells of varying hydrophobicity were mixed and exposed to predation, protozoa selectively removed the more hydrophobic cells [49]. Indeed, it has been reported that *V. cholerae* Δ*hapR* cells are more hydrophobic than isogenic WT cells [47].

VPS production is up regulated in Δ*hapR* mutant [18], [46], which forms flat undifferentiated biofilms (Fig. 4). It is also possible that this difference in biofilm architecture is the reason for selective removal of the Δ*hapR* mutant strain as it does not form microcolonies similar to the WT strain.

Interestingly, associational protection was observed in late stage mixed strain *V. cholerae* biofilms. CLSM image analysis shows that the majority of Δ*hapR* cells were eliminated while a small number of Δ*hapR* cells were located in the middle of WT microcolonies exposed to protozoan grazing. These remaining Δ*hapR* cells were protected from predation by the biofilm matrix produced by the WT. Associational protection was observed in protozoan grazing on mixed *P. fluorescens* CHA0 WT and isogenic QS mutant strains, where the grazing sensitive QS mutant was protected by the anti-protozoal mechanisms expressed by the WT when the relative abundance of the QS mutant was low [40]. The *V. cholerae* Δ*hapR* cells gained associational protection only when they were embedded within the WT microcolonies, indicating that the protective mechanism was physical protection and not due to the anti-protozoal factors released by the WT cells.

The bacterial-protozoal interaction has been suggested to be one of the oldest interactions between prokaryotic and eukaryotic organisms [50], and hence a major driving force in the evolution of pathogenesis by bacteria in the environment [51]. The data presented here demonstrate that *V. cholerae* possesses various biofilm mediated defensive mechanisms which allow it to survive grazing pressure. These findings highlight the role of biofilm as a protective environmental niche for the long term persistence of *V. cholerae*. In addition, some grazing resistant mechanisms expressed by *V. cholerae* are also involved in pathogenicity in humans [18], [52]. It may be hypothesised that many of the accessory virulence factors produced by *V. cholerae* biofilms have a role in increasing fitness in the environment rather than as ‘virulence factors’ specifically targeting humans during infection.
